# Performance of self-reported adherence to oral pre-exposure prophylaxis (PrEP) among HIV heterosexual serodiscordant couples in rural Uganda

**DOI:** 10.1186/1742-4690-9-S2-P213

**Published:** 2012-09-13

**Authors:** FM Kibengo, E Ruzagira, UM Bahemuka, D Katende, A Abaasa, B Barin, F Priddy, J Haberer, A Kampala

**Affiliations:** 1 Medical Research Council/Uganda Virus Research Institute, Entebbe, Uganda; 2 The EMMES Corporation, New York, NY, USA; 3 International AIDS Vaccine Initiative, New York, USA; 4 Massachusetts General Hospital Center for Global Health, Boston, MA, USA

## Background

Adherence is one of the main determinants of PrEP efficacy. Most PrEP studies applied subjective adherence measures, which often produce overestimates and problematic efficacy data interpretation; creating a need for more objective measures. This study examines self-reported adherence to oral PrEP compared to Medical Events Monitoring System (MEMS).

## Methods

Seventy-two HIV-uninfected partners (50% women) in Uganda were randomized to daily or intermittent (Monday, Friday and within 2 hours after sex, not exceeding 1 dose/day) oral emtricitabine/tenofovir or placebo in a 2:1:2:1 ratio for four months. Adherence was assessed monthly by MEMS and self-reported taken or missed doses by timeline follow-back calendar. MEMS data was adjusted for extra openings without pill removal and removal of multiple pills. Non-fixed days within intermittent regimen were classified as adherent/non-adherent based on self-reported sex by SMS. Adherence rates by taken/missed doses were compared to raw MEMS data using Spearman correlation.

## Results

Treatment and placebo groups were combined since adherence rates were similar. Daily raw MEMS adherence rate was significantly higher than fixed Intermittent rate (p=0.04) and post-coital dosing rate (p<0.0001). Raw MEMS data for daily and fixed intermittent dosing, poorly correlated with self-reported taken doses (r=0.14, p=0.42 and r=0.01, p=0.94, respectively) and missed doses (r=0.30, p=0.08 and r=0.07, p=0.69, respectively). Self-reported daily adherence had high sensitivity but only fair positive predictive value (PPV) and very poor specificity. Self-reported adherence to intermittent fixed dosing had fair sensitivity, PPV and negative predictive value (NPV), but poor specificity. Self-reported adherence to post-coital dosing had very good sensitivity and NPV but poor specificity.

## Conclusion

Median adherence for daily and intermittent fixed PrEP was high by objective and subjective measures, but poorly correlated. Adherence to post-coital dosing was poor and likely overestimated by self-report (possibly reflecting technical challenges of SMS). Self-reported adherence measures were highly sensitive but poorly specific.

